# High-Temperature Layered Modification of Mn_2_In_2_Se_5_

**DOI:** 10.3390/molecules30091904

**Published:** 2025-04-24

**Authors:** Ivan V. Chernoukhov, Anton D. Pyreu, Andrey N. Azarevich, Alexander N. Samarin, Alexey V. Bogach, Konstantin O. Znamenkov, Andrei V. Shevelkov, Valeriy Yu. Verchenko

**Affiliations:** 1 Department of Chemistry, Lomonosov Moscow State University, 119991 Moscow, Russia; 2 Prokhorov General Physics Institute of the Russian Academy of Sciences, 119991 Moscow, Russia

**Keywords:** vdW-chalcogenides, layered selenides, “225” family, frustrated system

## Abstract

Layered chalcogenides are interesting from the point of view of the formation of two-dimensional magnetic systems for relevant applications in spintronics. High-spin Mn^2+^ or Fe^3+^ cations with five unpaired electrons are promising in the search for compounds with interesting magnetic properties. In this study, a new layered modification of the Mn_2_In_2_Se_5_ compound from the A_2_B_2_X_5_ family (“225”) was synthesized and investigated. A phase transition to the polymorph with primitive trigonal lattice was recorded at a temperature of 711 °C, which was confirmed by simultaneous thermal analysis, X-ray powder diffraction at elevated temperatures, and sample annealing and quenching. The stability of Mn_2_In_2_Se_5_ in air at high temperatures was investigated by thermal gravimetric analysis and powder X-ray diffraction. The new polymorph of Mn_2_In_2_Se_5_ crystallizes in the Mg_2_Al_2_Se_5_ structure type, as revealed by the Rietveld refinement against powder X-ray diffraction data. The crystal structure can be viewed as a close-packing of Se anions, in which indium and manganese cations are enclosed inside tetrahedral and octahedral voids, respectively, according to the *A*_Mn_*B*_In_*CB*_In_*C*_Mn_*A*… sequence. Magnetization measurements reveal an antiferromagnetic-like transition at a temperature of 6.3 K. The same magnetic properties are reported in the literature for the low-temperature R-centered trigonal polymorph. An approximation by the modified Curie–Weiss law yields a significant ratio of |*θ*|/*T*_N_ = 28, which indicates strong magnetic frustration.

## 1. Introduction

Layered compounds possess crystal structures, which can be represented as blocks containing several atomic layers with relatively strong chemical bonds, while at the boundaries of blocks, only low-energy Van der Waals forces are present. Layered compounds are interesting for research for three main reasons: their functional properties, their use in the production of two-dimensional and pseudo-two-dimensional materials, and the possibility of chemical composition modification while preserving the overall structure [1,2]. Two-dimensional materials are unique in terms of their electronic and magnetic properties that find applications in developing spintronics technologies [3,4].

Often, the structure of ternary and more complex layered compounds inherits the base layers of binary representatives modified into larger blocks. Such families may contain a large number of representatives, which makes them a perfect playground for finding novel functional materials. Ternary families with a layered crystal structure include compounds with the stoichiometry of *AB*_2_*X*_4_ and *A*_2_*B*_2_*X*_5_, where *A* is a two-charge cation (usually Mg, Mn, Fe or Zn, Pb), *B* is a three-charge cation (for example, Al, Ga, In, Sb, Bi), and *X* is a chalcogen, S, Se or Te.

**Figure 1:**
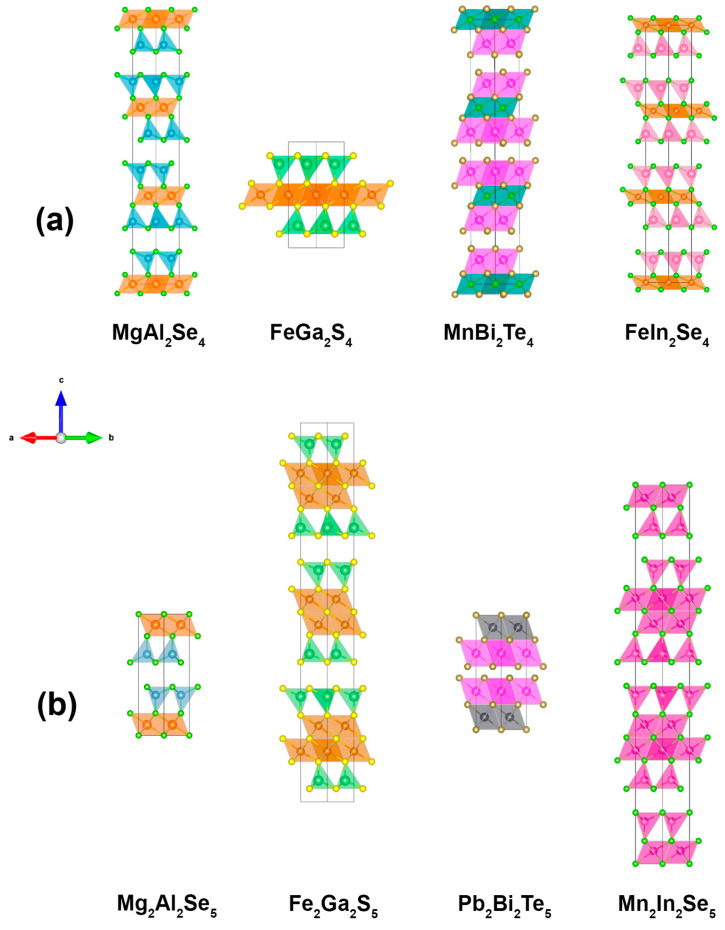
Crystal structure and unit cell of two families of homologues: (**a**) “124”—MgAl_2_Se_4_, FeGa_2_S_4_, MnBi_2_Te_4_, FeIn_2_Se_4_ (from left to right) and (**b**) “225”—Fe_2_Ga_2_S_5_, Mg_2_Al_2_Se_5_, Mn_2_In_2_Se_5_, Pb_2_Bi_2_Te_5_ (from left to right) [5,6,8,9,10,11,12]. The following color scheme is used: in the octahedral positions Mg—orange, Fe—brown, Bi—purple, Mn—green, Pb—gray, Mn-In –magenta and pink; in the tetrahedral positions Al—blue, Ga—green, In—pink, In-Mn –magenta and pink; in the anion positions S—yellow, Se—green, Te—golden.

The family of layered chalcogenides with *AB*_2_*X*_4_ (“124”) stoichiometry is represented by the following prototypes: MgAl_2_Se_4_ (MgAl_2_Se_4_, hR21, 166), FeGa_2_S_4_ (FeGa_2_S_4_, hP7, 164), MnBi_2_Te_4_ (MnBi_2_Te_4_, hR7, 166), and FeIn_2_Se_4_ (FeIn_2_Se_4_, hR7, 166) [5,6,7,8,9]. The crystal structures of all four representatives consist of blocks, where the two-charge cations octahedrally coordinated by chalcogen are located in the center of the block, and two outer layers of a three-charge cations enclose the block (Figure 1). Three-charge cations Al and Ga prefer tetrahedral coordination by chalcogen, while bismuth—octahedral. The unit cell of the FeGa_2_S_4_ structure type contain one block and one formula unit, while MgAl_2_Se_4_ and MnBi_2_Te_4_—three. The *A_2_B_2_X_5_* (“225”) family includes four main types of layered structures: Mg_2_Al_2_Se_5_ (Mg_2_Al_2_Se_5_, hP9, 164), Fe_2_Ga_2_S_5_ (Fe_2_Ga_2_S_5_, hP18, 194), Mn_2_In_2_Se_5_ ((Mn_0.5_In_0.5_)_4_Se_5_, hR27, 166), and Pb_2_Bi_2_Te_5_ (Pb_2_Bi_2_Se_5_, hP9, 164) [6,10,11,12]. The block in the structure of these compounds is similar to the block of the homologous “124” family, but with one additional layer of two-charge cations in the center. The “124” and “225” families demonstrate unique structural flexibility, where both the alternation of octahedral and tetrahedral sites and the mixed occupation of crystallographic positions are possible due to the close values of ionic radii of A and B cations. For example, as many as 16 layered polytypes were observed for the ZnIn_2_S_4_ compound [13,14]. The “124” and “225” compounds, which are based on a transition metal with a partially filled 3D shell, attract special interest due to their unique magnetic properties, including strong frustration on a triangular lattice [15,16,17], and nearly two-dimensional antiferromagnetic ordering [18].

Previously in the “124” and “225” families, we discovered new layered Mn-based compounds, MnAl_2_S_4_, MnAl_2_Se_4_, Mn_2_Al_2_Se_5_, and Mn_2_Ga_2_S_5_, with MgAl_2_Se_4_- and Mg_2_Al_2_Se_5_-type structures [19,20]. Interestingly, MnIn_2_Se_4_ [21] and Mn_2_In_2_Se_5_ [11,22,23] also possess layered crystal structures, where the latter crystallizes in the *R*−3*m* space group (in the context of this work, this polymorph is indicated as “R-phase”). This R-phase demonstrates strong magnetic frustration and a significant spin-glass contribution at low temperatures [11,22,23]. In this study, we revisited the Mn-In-Se ternary system and surprisingly found interesting phase relations, which are not in agreement with those reported recently [11,22,23]. Particularly, we found that the reaction of MnIn_2_Se_4_+MnSe→Mn_2_In_2_Se_5_ yields a new polymorphic modification of Mn_2_In_2_Se_5_, which fits the “225” family well. Here, we present the synthesis, crystal structure, oxidation resistance, and magnetic properties of the new high-temperature layered polymorph of Mn_2_In_2_Se_5_.

## 2. Results and Discussion

### 2.1. Synthesis of Polycrystalline Mn_2_In_2_Se_5_

Numerous syntheses of polycrystalline samples of Mn_2_In_2_Se_5_, accompanied by careful analysis of phase composition and Le Bail fitting of powder X-ray diffraction patterns, clearly indicate the formation of non-single-phase products, where MnSe is the most probable admixture. To optimize synthetic conditions, the annealing temperature was varied in the range between 700 °C and 1100 °C, the annealing time was increased up to 240 h, and intermediate grinding was introduced. Also, samples were pressed into pellets and the use of crucibles was tested to reduce possible side reactions. All these precautions, as well as the synthesis from the binary precursors MnSe and In_2_Se_3_, yielded the same non-single-phase composition, indicating that either the starting ratio of elements should be non-stoichiometric for the formation of the “Mn_2_In_2_Se_5_” phase, or this phase undergoes decomposition upon cooling. According to a recent report, Mn_2_In_2_Se_5_ decomposes peritectically at 920 °C; however, no other reactions were observed below the decomposition temperature [22].

### 2.2. Elemental and Phase Composition and Thermal Analysis of Mn_2_In_2_Se_5_

**Figure 2:**
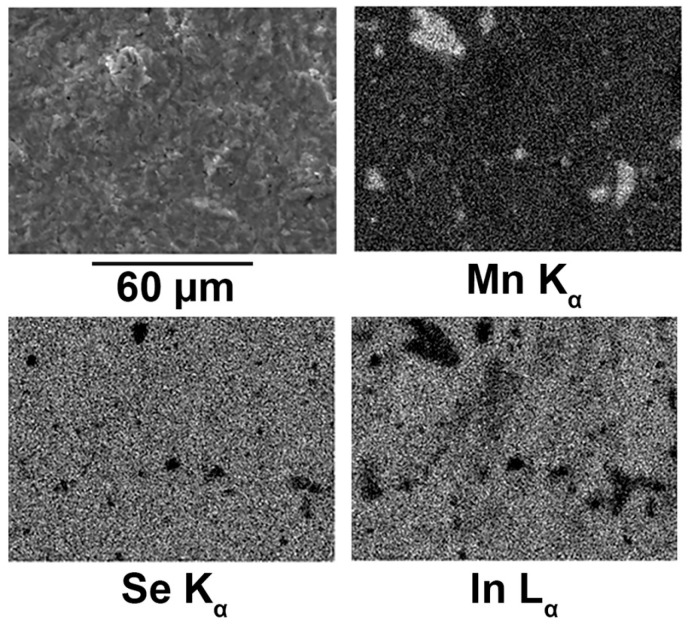
Energy-dispersive X-ray maps of Mn, Se, and In collected across the surface of pressed pellet of polycrystalline Mn_2_In_2_Se_5_.

The elemental composition and its uniformity were studied by scanning electron microscopy and energy-dispersive X-ray spectroscopy. Elemental maps registered across the surface of a pressed pellet clearly indicate areas with an increased fraction of manganese and a reduced content of indium, which correspond to the MnSe admixture (Figure 2). However, in the rest of the sample, the elements are distributed evenly, and the average composition of Mn_2.3(2)_In_2.1(1)_Se_4.7(2)_ is in quantitative agreement with the starting one. Obviously, the composition of the main phase does not deviate from the stoichiometric ratio; thus, we expect side reactions and more complex phase relations, which take place during the cooling of the polycrystalline sample from the annealing temperature.

**Figure 3:**
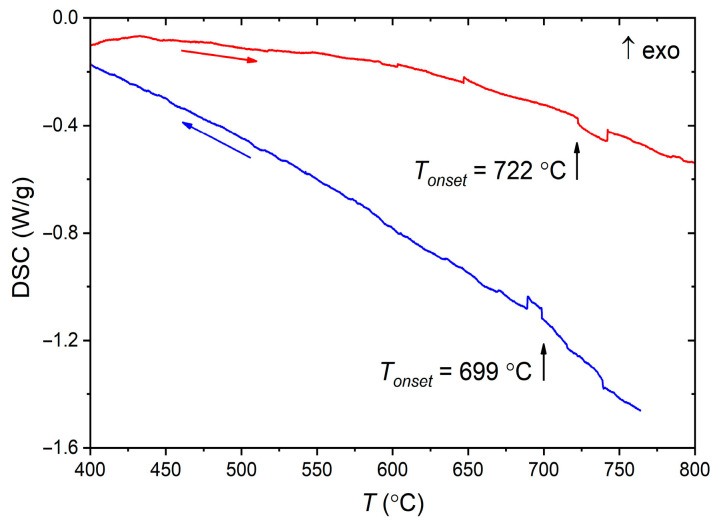
Differential scanning calorimetry of Mn_2_In_2_Se_5_ in the mid-temperature range. The heating and cooling curves are shown in red and blue, respectively.

Polycrystalline Mn_2_In_2_Se_5_ was studied by differential scanning calorimetry in the mid-temperature range of 400–800 °C (Figure 3). The onset of a weak endothermic peak was observed under heating. This peak demonstrates reproducible behavior and slight temperature hysteresis. The onset temperatures of 722 °C and 699 °C during heating and cooling, respectively, correspond to the average temperature of the transition of 711 °C. To investigate this transition, we measured powder X-ray diffraction (PXRD) patterns at various elevated temperatures.

**Figure 4:**
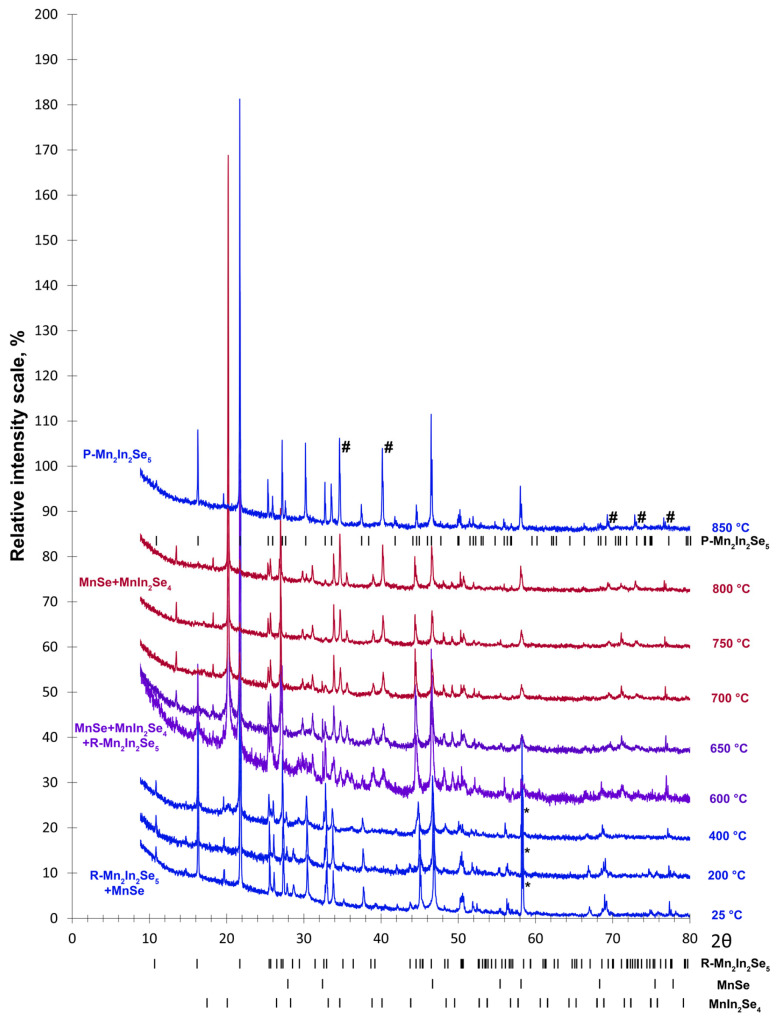
Powder X-ray diffraction patterns of Mn_2_In_2_Se_5_ at various elevated temperatures. Mn_2_In_2_Se_5_ and MnIn_2_Se_4_ phases are shown in blue and red, respectively. Black marks show the positions of peaks. The instrumental peak is indicated by asterisk. The new unknown phase formed at 850 °C is indicated by the hash symbols.

As already discussed, the as-prepared polycrystalline sample of Mn_2_In_2_Se_5_ is not single-phase and contains the admixture of MnSe. All reflections of Mn_2_In_2_Se_5_ at room temperature can be indexed using the R-centered trigonal unit cell with *a* = 4.02381(8) Å and *c* = 48.858(1) Å, in perfect agreement with the previous reports [11,22,23]. The main peak of MnSe is located at 2*θ* = 32.9° on the room-temperature PXRD pattern (Figure 4). This mixture of phases persists upon heating up to 400 °C, and then Mn_2_In_2_Se_5_ decomposes to MnIn_2_Se_4_. The main peak of MnIn_2_Se_4_ is located at 2*θ* = 20.2°. In the temperature range of 600–700 °C MnIn_2_Se_4_ and Mn_2_In_2_Se_5_ coexist, while at 750–800 °C the mixture of MnIn_2_Se_4_ and MnSe was observed. It should be noted that the low-temperature decomposition of Mn_2_In_2_Se_5_ into MnIn_2_Se_4_ and MnSe is not accompanied by any peak on the DSC curve. Presumably, this decomposition goes gradually in the temperature range, where both Mn_2_In_2_Se_5_ and MnIn_2_Se_4_ coexist. Finally, the reaction of MnIn_2_Se_4_+MnSe→Mn_2_In_2_Se_5_ takes place at high temperatures above 800 °C, and the target compound Mn_2_In_2_Se_5_ is formed as the main phase again at 850 °C. Surprisingly, the reflections of Mn_2_In_2_Se_5_ at a high temperature correspond to a novel unit cell, which is primitive trigonal or hexagonal with *a* = 4.0742(1) Å and *c* = 16.4671(5) Å at 850 °C. Thus, the reaction of MnIn_2_Se_4_+MnSe→Mn_2_In_2_Se_5_ yields a new polymorph of the target compound, which will be indicated as “P-phase” later in the text. Moreover, the formation of Mn_2_In_2_Se_5_ at high temperatures is accompanied by the appearance of new peaks, which are marked by the hash symbols in Figure 4. These peaks can be indexed using a primitive trigonal or hexagonal unit cell with lattice parameters of *a* = 8.990(3) Å and *c* = 6.912(3) Å, indicating the formation of an unknown X phase in the Mn-In-Se system.

Combining the results of differential scanning calorimetry and PXRD at elevated temperatures, the observed effect at 711 °C presumably corresponds to the formation of Mn_2_In_2_Se_5_ according to the reaction, MnIn_2_Se_4_+MnSe→Mn_2_In_2_Se_5_, while its endothermic nature is connected with the fact that the Mn_2_In_2_Se_5_ and MnIn_2_Se_4_ homologous compounds possess similar layered crystal structures. Thus, the formation reaction may be endothermic in this case. PXRD measurements reveal a slightly higher temperature above 800 °C for this reaction than differential scanning calorimetry.

The results of high-temperature PXRD measurements were corroborated by standard ampule synthesis. A polycrystalline stoichiometric sample of Mn_2_In_2_Se_5_ was annealed at 900 °C for 60 h, and the ampule was subsequently quenched in cold water. As a result, the novel P-phase was stabilized at room temperature with only a tiny admixture of MnSe. No traces of the X phase were observed in this case. At the same time, the P-phase reproducibly transforms into the R-phase upon repeated annealing, as well as slowly decomposing into the mixture of MnIn_2_Se_4_ and MnSe under prolonged annealing at 200 °C or 300 °C.

### 2.3. Oxidation of Mn_2_In_2_Se_5_

**Figure 5:**
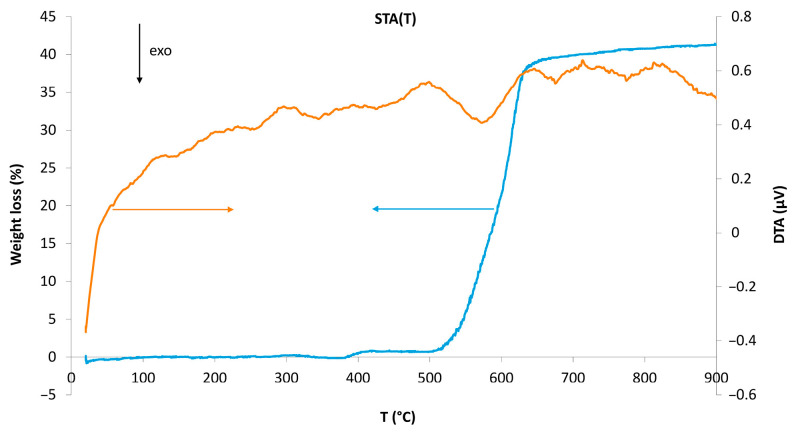
Combined thermal analysis of Mn_2_In_2_Se_5_ in air atmosphere. The curves of the differential scanning calorimetry and the weight loss are shown in orange and blue, respectively.

Chalcogenides, especially sulfides and selenides, may be unstable in air and react with oxygen or water vapor. Also, oxygen atoms may be present in the crystal structure replacing S or Se. To avoid possible inaccuracies in the chemical composition related to the reaction with oxygen and water, we investigated various aspects of the stability of Mn_2_In_2_Se_5_ in air (Figure 5). Thermal gravimetric analysis in ambient air was used to investigate the oxidation process. Combined thermal analysis shows a single-stage mass loss from 500 °C to 600 °C accompanied by a weak exothermic effect. The measured mass loss of 41.4% is in good agreement with the following oxidation equation:

(1) $$6{Mn}_{2}{In}_{2}{Se}_{5}+47O_{2}\;\overset{T}{\to }\;4{Mn}_{3}O_{4}+6{In}_{2}O_{3}+30SeO_{2}↑,$$

**Figure 6:**
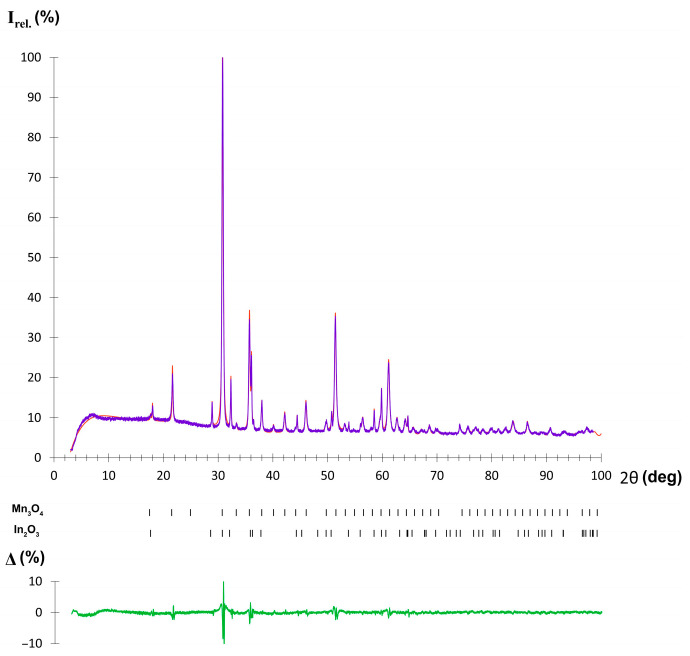
Phase composition of Mn_2_In_2_Se_5_ oxidation products after thermal analysis. The experimental data are shown by the purple dots, and the theoretical pattern is presented by the orange line. Black marks show the positions of reflections. The difference curve is shown as a green line.

The reaction products were analyzed by powder X-ray diffraction (Figure 6). Phase analysis clearly indicates the presence of Mn_3_O_4_ and In_2_O_3_ in good agreement with the oxidation model proposed in Equation (1). Overall, Mn_2_In_2_Se_5_ demonstrates good stability in ambient air, where no bulk oxidation occurs at temperatures at least below 500 °C.

### 2.4. Crystal Structure of the New Polymorph of Mn_2_In_2_Se_5_

**Figure 7:**
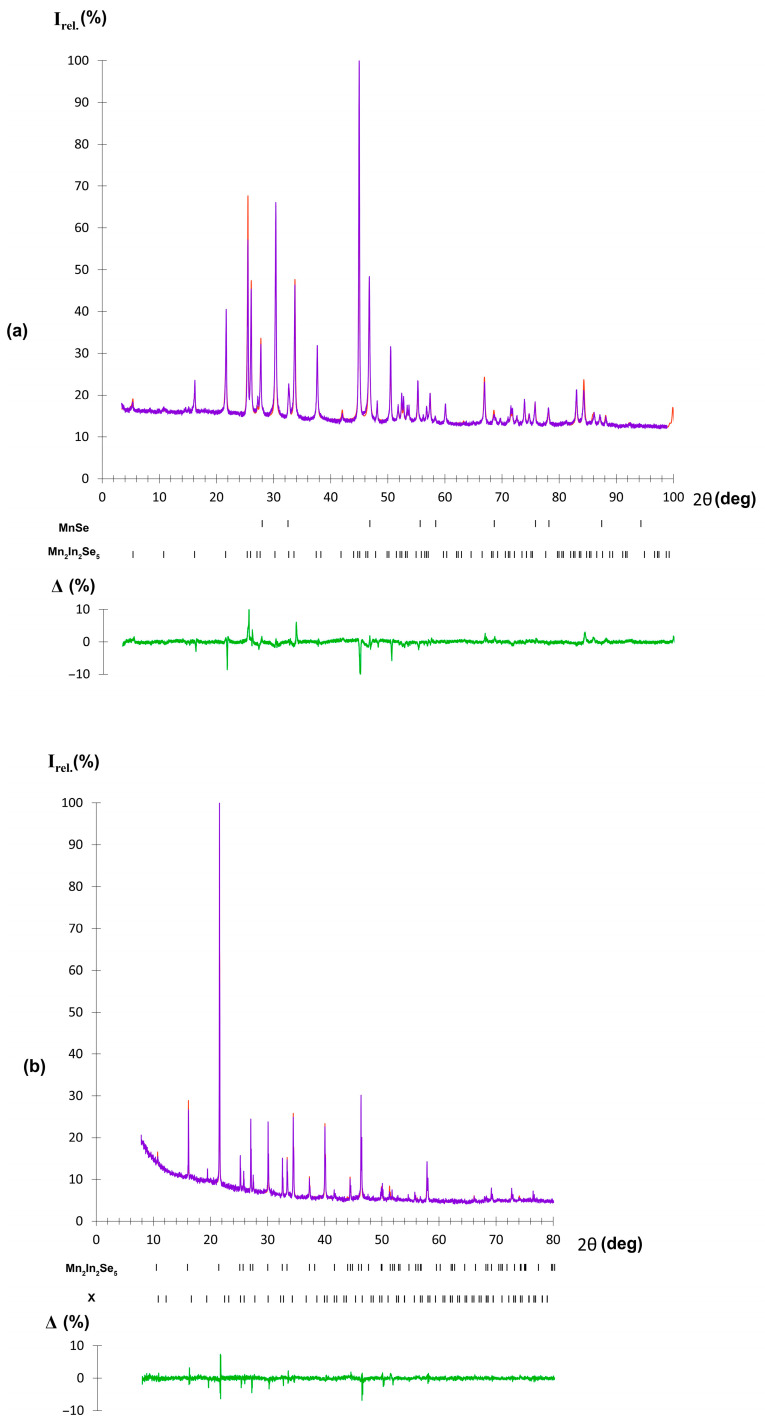
Powder X-ray diffraction patterns of the P-phase of Mn_2_In_2_Se_5_ at room temperature (**a**) and at 850 °C (**b**). The experimental data are shown by the purple dots, and the theoretical pattern is presented by the orange line. Black marks show the positions of reflections. The difference curve is shown as a green line.

The crystal structure of the new layered polymorph of Mn_2_In_2_Se_5_ was obtained by the Rietveld refinement against the high-temperature PXRD data, as well as by using the room-temperature PXRD pattern of polycrystalline sample quenched from 900 °C. Both PXRD patterns can be indexed in the primitive trigonal unit cell. For example, the high-temperature data yield unit cell parameters of *a* = 4.0742(1) Å and *c* = 16.4671(5) Å, which are close to those of Mg_2_Al_2_Se_5_. The latter is a prototype structure for a number of layered “225” compounds, including Mn_2_Ga_2_S_5_ and Mn_2_Al_2_Se_5_ [6,20]. Therefore, the unit cell parameters of the crystal structure of Mg_2_Al_2_Se_5_ were used as a starting model for the refinement. Mn and In atoms were placed on the Mg and Al sites, respectively. Data collection and refinement details are given in Table 1. The obtained parameters of atomic positions and selected interatomic distances are provided in Table 2 and Table 3, respectively. The experimental and calculated PXRD patterns are shown in Figure 7.

**Figure 8:**
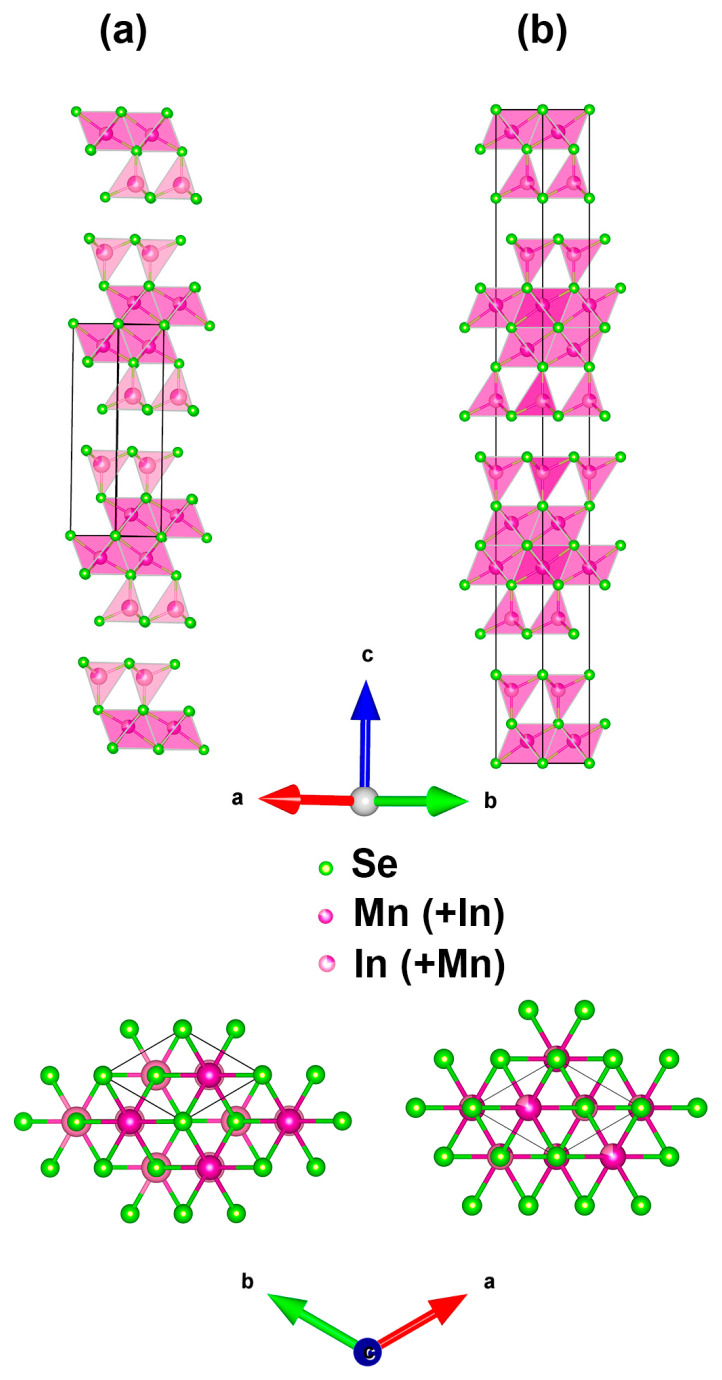
Crystal structure and unit cell of the P-phase (**a**) and R-phase (**b**) of Mn_2_In_2_Se_5_.

According to the refinement, the new modification of Mn_2_In_2_Se_5_ possesses a layered crystal structure, which is shown in Figure 8a. The structure can be described by blocks with the Se atoms on boundaries, and the adjacent blocks are separated by van der Waals gaps. Each block is formed by an alternation of closely packed layers of Se anions, where Mn and In cations populate octahedral and tetrahedral voids, respectively. The following sequence of layers describes a single structural block, *A*_Mn_*B*_In_*CB*_In_*C_M_*_n_*A*..., with two-layered packing of Mn and In between the *CBC*... and *BCB*... layers, respectively. The crystal structure of the R-phase, which is shown in Figure 8b, is constructed in a similar way, but with a more complex arrangement of anionic layers: *A*_Mn_*B*_In_*AC*_In_*B*_Mn_*C*_Mn_*A*_In_*CB*_In_*A*_Mn_*B*_Mn_*C*_In_*BA*_In_*C*_Mn_*A*… with three-layered packing *CABC*… for Mn and a mixed *BBAACCB*… for In. Nevertheless, both the P- and R-phases possess similar atomic environments regarding Mn and In cations, as revealed by the observed interatomic distances (Table 3). If the occupancy is not taken into account, this new structure is identical to Mg_2_Al_2_Se_5_ with the corresponding substitution of cations to Mn and In. The crystal structure of the P-phase of Mn_2_In_2_Se_5_ is closely related to the Mg_2_Al_2_Se_5_ structure type. It has the same coordination environment of atoms, content of the unit cell content, and connection of the adjacent blocks. At the same time, the Pb_2_Bi_2_Te_5_ structure type has different environments of cations, while Fe_2_Ga_2_S_5_ and the R-phase of Mn_2_In_2_Se_5_ possess larger unit cells.

The refinement of displacement parameters clearly indicates that both Mn and In are mixed in the octahedral and tetrahedral positions. At room temperature, this mixing is moderate: the octahedral sites contain 70% of Mn, while tetrahedral—70% of In (Table 2). At high temperatures, antisite defects are largely present in the crystal structure, yielding the uniform distribution of Mn and In across octahedral and tetrahedral sites. Remarkably, the octahedral position has a large value displacement parameter at 850 °C, indicating possible structural instability in the vicinity of peritectic decomposition. The interatomic distances, which are listed in Table 3, point at the pronounced temperature expansion at 850 °C, too.

### 2.5. Magnetic Properties of Mn_2_In_2_Se_5_

**Figure 9:**
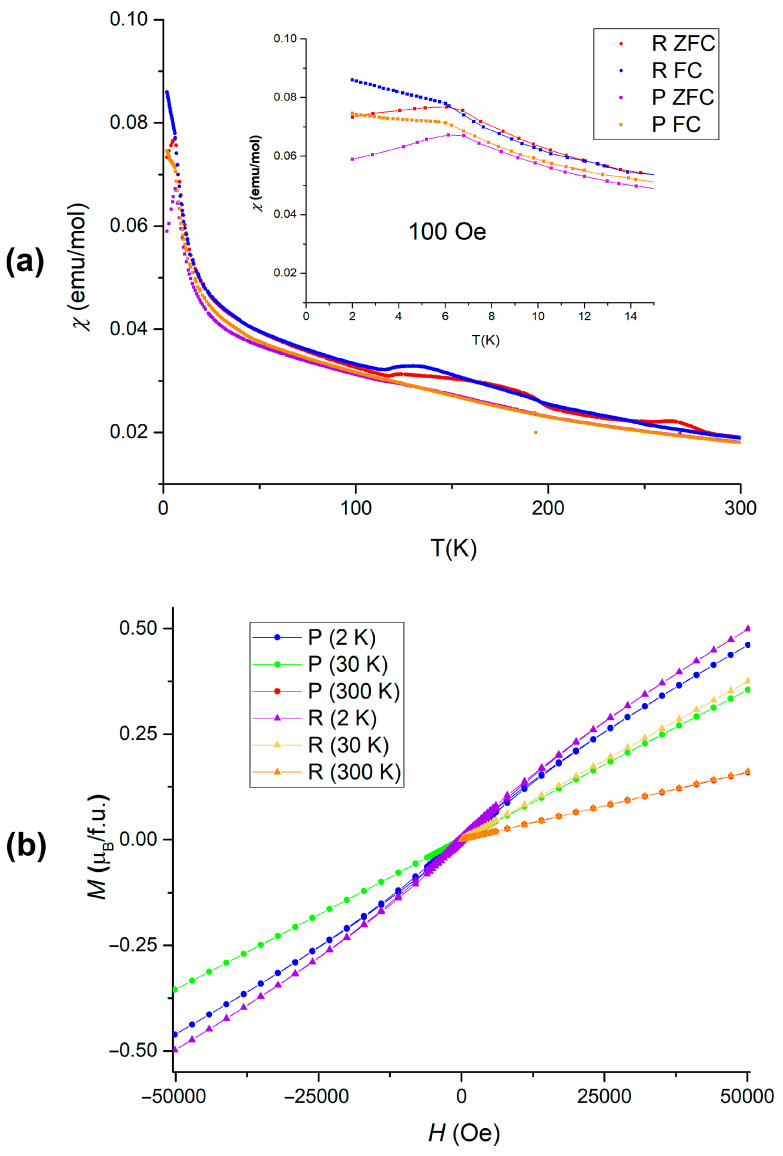
Magnetic susceptibility of P-phase and R-phase of Mn_2_In_2_Se_5_ measured in the zero-field-cooling (ZFC) and field-cooling (FC) conditions (**a**). Magnetization measurements at various temperatures (**b**).

Given the investigated phase transformations and established synthesis protocols, two polymorphs of Mn_2_In_2_Se_5_ were obtained independently with a minimal amount of admixtures. The magnetic properties of the P-phase and R-phase of Mn_2_In_2_Se_5_ were probed by DC magnetization measurements, which are shown in Figure 9. According to the magnetic susceptibility data, both samples demonstrate Curie–Weiss-type paramagnetic behavior at elevated temperatures. An onset of antiferromagnetic-like transition is visible at a temperature of 6.3 K for the P-phase and at 6 K for the R-phase, which is suppressed by an external magnetic field. This transition is accompanied by a slight hysteresis of magnetization at 2 K, while at higher temperatures the magnetization follows simple paramagnetic behavior.

The magnetic susceptibility of the R-phase measured in 100 Oe magnetic field reveals the presence of impurity in the temperature range of 100–300 K, which is extremely characteristic of MnSe [24]. The contribution of impurity is suppressed by the increasing magnetic field. MnSe is always formed as a result of the partial decomposition of the main phase when attempting to obtain the R-phase at low temperatures, and it is observed by PXRD phase analysis, as indicated previously in the text. Otherwise, the magnetic properties of the P-phase and R-phase perfectly coincide, in agreement with the fact that both polymorphs are based on the same structural block (Figure 8).

**Figure 10:**
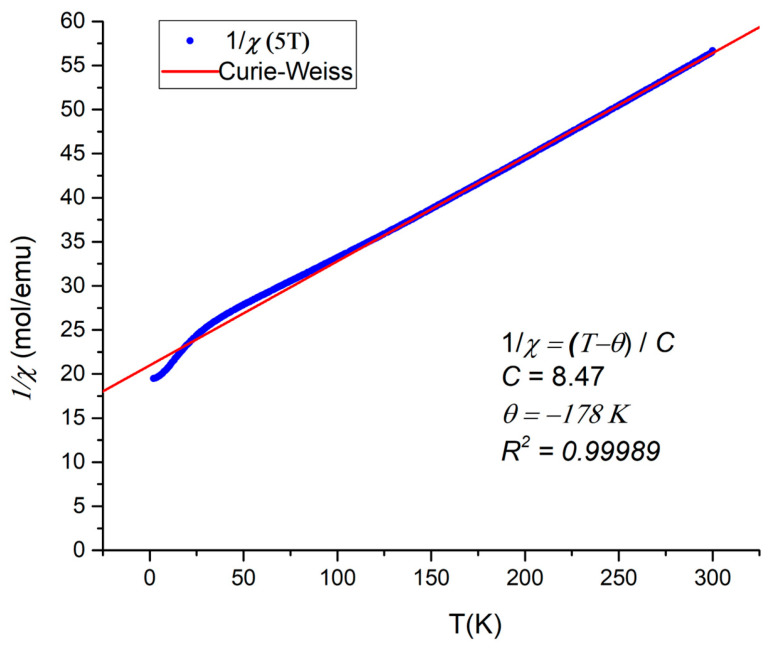
Inverse magnetic susceptibility of P-phase of Mn_2_In_2_Se_5_ in 5 T magnetic field (blue dots) and its fit by Curie–Weiss law (red line).

An approximation of the high-temperature inverse magnetic susceptibility of the P-phase by the Curie–Weiss law yields a Weiss temperature of *θ* = −180 K with a ratio of |*θ*|/*T*_N_ = 28 (Figure 10). The observed negative value of *θ* indicates the strong exchange interaction of the antiferromagnetic nature between the neighboring paramagnetic centers, while large |*θ*|/*T*_N_ points at the significant frustration in the system. Thus, the low dimensionality and large frustration index of the P-phase are similar to those reported recently for the R-phase [23]. The effective magnetic moment of µ= 4.12 µ_B_ per Mn atom turns out to be significantly lower than expected for Mn^2+^ = 5.92 µ_B_. The same deviation was observed in the isostructural compound, Mn_2_Ga_2_S_5_ [20].

## 3. Materials and Methods

For the synthesis of Mn_2_In_2_Se_5_, Mn (plates, 99%, Sigma-Aldrich, Steinheim, Germany), In (lump, 99.99%, Sigma-Aldrich, Steinheim, Germany), and Se (granules, 99.999%, Sigma-Aldrich, Steinheim, Germany) were used. All operations with precursors and samples were performed in an argon-filled glove box (Spectro-systems, p(H_2_O, O_2_) < 1 ppm). The precursors were weighed in a stoichiometric molar ratio with an accuracy of 0.1 mg. Before annealing, they were placed in a quartz ampule, which was evacuated to a residual pressure of 5 × 10^−3^ mbar and flame-sealed. The series of samples were prepared using annealing temperatures of 973 K, 1073 K, 1173 K, and 1273 K, each for 5 days with intermediate grinding. Additionally, pellet pressing and the use of a crucible inside an ampoule were tested. Samples were pressed into a pellet with a diameter of 6 mm at a pressure of 1200 kgf/cm^2^. However, these samples exhibited multiphase composition based on the R-phase. To obtain the P-phase, the stoichiometric mixture of precursors enclosed inside an evacuated quartz ampule was annealed at 1173 K for 60 h and subsequently quenched in water.

Samples were studied by differential scanning calorimetry using a simultaneous thermal analyzer STA 449 F3 Jupiter in a high-purity argon flow of 240 mL/min. During the first measurement, the sample was heated twice to 800 °C and cooled to 400 °C at a rate of 10 °C/min. Secondly, the data between 600 °C and 900 °C were acquired at a lower rate of 3 °C/min. TG-DTA data were obtained on a Derivatograph Q-1500 D in air. The oxidation process was studied by heating the sample to 900 °C at a rate of 10 °C/min.

A phase composition study and crystal structure refinements were performed by powder X-ray diffraction. Room-temperature data were obtained on a Huber G670 Guinier camera (Cu Kα_1_ radiation, Ge_111_ monochromator, image plate detector). The samples were enclosed between two mylar films and fixed in a sample holder in an argon-filled glove box. High-temperature data were acquired using a Bruker D8 Advance diffractometer (Cu X-ray source, no monochromator, LYNXEYE detector) equipped with the XRK900 temperature chamber. Measurements were performed in a flow of high-purity argon at temperatures of 200, 400, 600, 650, 700, 750, 800 and 850 °C. The experimental and refinement details, parameters of atomic positions, and selected interatomic distances are given in Table 1, Table 2 and Table 3, respectively. Indexing was performed using the VISSER algorithm in the WinXPow program (version 2.25). The crystal structure was refined using the Jana2006 program [25].

Images of pellets, mapping, and analysis of the elemental composition were obtained using a scanning electron microscope (SEM) JSM JEOL6490-LV with an energy-dispersive X-ray (EDX) detection system INCA x-Sight at an accelerating voltage of 20 kV.

The magnetization of polycrystalline samples was measured using a Magnetic Properties Measurement System (Quantum Design, MPMS-XL5 SQUID). The measurements were performed in zero-field-cooling and field-cooling conditions. Temperature dependences were measured from 2 K to 300 K in magnetic fields of 0.01 T and 5 T and field dependences were measured at temperatures of 2, 30, and 300 K, while sweeping the magnetic field from −5 T to 5 T.

## 4. Conclusions

Mn_2_In_2_Se_5_ is a layered van der Waals compound based on the magnetic Mn^2+^ species. Its layered crystal structure is formed by alternating hexagonal close-packing layers of Se. As a result, the Mn atoms feature a perfect triangular arrangement within the two adjacent layers of octahedral voids in the center of the van der Waals block. This triangular magnetic system can be easily prepared in a two-dimensional fashion due to low-energy van der Waals bonds between the structural blocks. Thus, Mn_2_In_2_Se_5_ is a perfect system to study strong frustration on a triangular lattice in two dimensions. In this study, we carefully developed synthesis protocols to obtain high-quality polycrystalline samples of the new high-temperature polymorph of Mn_2_In_2_Se_5_. This polymorph is stable against oxidation up to 400 °C; thus, stability in two-dimensional nanomaterials is expected, too. The bulk material demonstrates strong antiferromagnetic coupling between the paramagnetic centers, two-dimensionality, and large frustration, as revealed by magnetization measurements.

## Figures and Tables

**Table 1:** Details of data collection and structure refinement for the P-phase of Mn_2_In_2_Se_5_.

Parameter	Value	
sample	high-temperature	quenched from 900 °C
composition	Mn_2_In_2_Se_5_	
formula weight (g/mol)	734.31	
diffractometer	Bruker D8 Advance	Huber G670
detector	LYNXEYE	image plate
radiation	Cu K*α*_1,2_	Cu K*α*_1_
wavelength (Å)	1.5419	1.5406
crystal system	trigonal	
space group	*P*$\bar{3}$*m*1	
Z	1	
unit cell parameters		
*a* (Å)	4.0742(1)	4.02535(5)
*c* (Å)	16.4671(5)	16.3503(3)
*V* (Å^3^)	236.72(1)	229.438(6)
temperature (K)	1123	298
*ρ*_calc_ (g/cm^3^)	5.15	5.31
μ (cm^−1^)	81.0	83.6
2*θ* range (deg)	5–80	3–100
*R*_p_	0.0404	0.0257
*wR*_p_	0.0545	0.0379
*R*_obs_	0.1124	0.0923
*wR*_obs_	0.0937	0.1158
*R*_all_	0.1278	0.0987
*wR*_all_	0.0967	0.1159
GOF	1.19	3.17
parameters	33	31
constraints	7	7
residual peaks (e^−^/Å^3^)	2.81/−3.30	4.71/−3.29

**Table 2:** Parameters of atomic positions for the crystal structure of P-phase of Mn_2_In_2_Se_5_.

(a)	P-Phase of Mn_2_In_2_Se_5_ (850 °C)					
Label	Symmetry	*x*	*y*	*z*	Occupancy	*U*_iso_ (Å^2^)
Mn1	3*m*	1/3	2/3	0.1048(1)	0.5Mn + 0.5In	0.150(2)
In1	3*m*	1/3	2/3	0.6631(2)	0.5In + 0.5Mn	0.032(2)
Se1	$\bar{3}$ *m*	0	0	0	1	0.069(6)
Se2	3*m*	1/3	2/3	0.4014(3)	1	0.062(4)
Se3	3*m*	1/3	2/3	0.8145(3)	1	0.023(3)
(**b**)	**P-Phase of Mn_2_In_2_Se_5_ (room temperature)**					
**Label**	**Symmetry**	***x***	***y***	***z***	**Occupancy**	***U*_iso_ (Å^2^)**
Mn1	3*m*	1/3	2/3	0.1025(1)	0.704(2)Mn + 0.296In	0.0186(9)
In1	3*m*	1/3	2/3	0.66222(8)	0.704(2)In + 0.296Mn	0.0147(4)
Se1	$\bar{3}$ *m*	0	0	0	1	0.0108(8)
Se2	3*m*	1/3	2/3	0.4017(1)	1	0.0171(7)
Se3	3*m*	1/3	2/3	0.8201(1)	1	0.0193(8)

**Table 3:** Selected interatomic distances in the crystal structure of Mn_2_In_2_Se_5_.

P-Phase of Mn_2_In_2_Se_5_ (850 °C)		
Central Atom	Neighbor Atom	Distance (Å)
Mn1	Se1 (×3)	2.917(1)
	Se3 (×3)	2.702(2)
In1	Se2 (×3)	2.581(2)
	Se3 (×1)	2.492(5)
P-phase of Mn_2_In_2_Se_5_ (room temperature)		
Central atom	Neighbor atom	Distance (Å)
Mn1	Se1 (×3)	2.865(1)
	Se3 (×3)	2.647(1)
In1	Se2 (×3)	2.5483(9)
	Se3 (×1)	2.581(2)
R-phase of Mn_2_In_2_Se_5_ [11]		
Central atom	Neighbor atom	Distance (Å)
Mn1	Se1 (×3)	2.8508(7)
	Se3 (×3)	2.6496(8)
In1	Se2 (×3)	2.5840(6)
	Se3 (×1)	2.516(1)

## Data Availability

The corresponding author will provide data on request due to necessary explanation.
